# A comparison of food portion size estimation methods among 11–12 year olds: 3D food models vs an online tool using food portion photos (Intake24)

**DOI:** 10.1186/s40795-021-00415-5

**Published:** 2021-05-06

**Authors:** Jennifer Bradley, Maisie K. Rowland, John N. S. Matthews, Ashley J. Adamson, Suzanne Spence

**Affiliations:** 1 Human Nutrition Research Centre, Population Health Sciences Institute, Faculty of Medical Sciences, Newcastle University, Newcastle upon Tyne, NE2 4HH UK; 2 School of Mathematics, Statistics and Physics, Newcastle University, Newcastle upon Tyne, NE1 7RU UK

**Keywords:** Dietary assessment, Portion size estimation, Children, Adolescents, Intake24, Food models

## Abstract

**Background:**

Technology has advanced bringing new cost-effective methods to measure food intake. The aim of the study was to compare food and drink portion estimates from a traditional portion estimation method using 3D food models with portion estimates using an online dietary recall tool, Intake24.

**Methods:**

11-12 year old children were recruited from secondary schools in Newcastle upon Tyne. Each pupil completed a two-day food diary followed by an interview during which pupils estimated food portion sizes using a range of 3D food models. They also completed Intake24 for the same 2 days. Bland Altman analyses were used to compare mean intake for each method.

**Results:**

Seventy pupils completed both portion estimation methods. There was good agreement in food weight estimations between the two methods (geometric mean ratio 1.00), with limits of agreement ranging from minus 35% to plus 53%. Intake24 provided estimates of energy intake that were 1% lower on average than estimates of energy intake using the food models. Mean intakes of all macro and micronutrients using Intake24 were within 6% of the food model estimates.

**Conclusions:**

The findings suggest that there was little difference in portion estimations from the two methods, allowing comparisons to be made between Intake24 data and food diary data collected from same age pupils using 3D food models in previous years.

## Introduction

One in three children starting secondary school in the UK are overweight and/or obese [1]. Intakes of free sugars and saturated fat are above recommended levels in this age group, and intakes of fibre, fruit and vegetables, and oily fish are below UK recommendations [2]. Unhealthy dietary habits are a major contributing factor to the risk of non-communicable diseases (NCDs) [3, 4]. Understanding the dietary habits of children and adolescents is vital especially as they gain increasing autonomy over their food choices [5], and there is evidence of dietary patterns tracking from adolescence into adulthood [6, 7].

One such study has been instrumental in understanding dietary intakes in 11–12 year olds. The Northumberland Middle Schools study is a cross-sectional dietary study led by Newcastle University. Dietary data has been collected from 11 to 12 year olds from schools in the Morpeth and Ashington areas of Northumberland in 1980 (*n* = 405), 1990 (*n* = 379), 2000 (*n* = 424), and 2010 (*n* = 295) (collectively known as the ASH11 studies). Findings from the studies have been previously published [8–13] and evidence has been important in identifying required changes in children’s diet and in contributing to school food policy. The School Food Plan was commissioned by the Department for Education and published in 2013 [14]. Findings from prior work highlighted school food has potential to improve children’s diets [15]. The report set out recommendations which aimed to improve school food culture and access to good food [13].

A traditional paper-based three-day estimated food diary has been used to collect dietary data at two time points for each study year, each followed by an interview with a trained researcher to clarify the information recorded [13]. During the interview three-dimensional (3D) food models were used to aid portion size estimation along with food portion photographs [16]. However, the last 40 years have seen an increase in the variety of foods and drinks available both in and outside the home, meaning the types of foods consumed have changed since the first ASH11 study in 1980 [17]. In addition, technology has advanced bringing new cost-effective methods to measure food intake [18]. Intake24 is an online 24-h dietary recall tool developed by researchers at Newcastle University [19]. It is a validated tool developed for users aged 11 years upwards [20], and is based on the multiple pass 24-h recall method [21]. Intake24 contains a database of over 2500 foods which are linked to nutrient composition codes. Portion size estimation is aided by a series of food portion photographs which have been validated previously [22]. Intake24 can be completed on a computer, laptop, tablet or mobile phone.

For the next ASH11 study, due to commence in the 2020/2021 academic year, 3D food models will be replaced with Intake24 to estimate portion size. The food model method is a time-consuming process, requiring transport of the food models to school and arrangement of the models for each participant. The use of Intake24 will streamline data collection, as it can be accessed via a website on a computer or laptop in school, therefore minimal equipment is required. The integrated food list and portion estimation databases in Intake24 also remove the need for manual coding and data entry; a process which has taken a considerable amount of time and resource in previous ASH11 studies due to the large numbers of pupils taking part (approximately 300 pupils in the 2010 study each completing two food three-day diaries). The range of portion size photos available in Intake24 is much greater than the 3D food models, and these can be added to and updated when required. However, it is imperative that the potential impact of a change in portion estimation method is examined before the next ASH11 study.

The aim of the present study was to compare the original method of food and drink portion estimation using 3D food models (reference method) with portion estimates using the Intake24 method (test method). The objectives of the study were to compare individual mean daily food weight intakes, nutrient intakes and food group intakes estimated using Intake24 with intakes estimated using 3D food models in a sample of 11–12 year olds from secondary schools in Newcastle upon Tyne. Levels of agreement were examined to determine whether the agreement between the two methods was sufficient to support replacing the use of 3D food models in future ASH11 studies.

## Materials and methods

Ethical approval for the study was granted by Newcastle University ethics committee (application number 1604/6905/2018). All methods were carried out in accordance with relevant guidelines and regulations. Four secondary schools in Newcastle upon Tyne were recruited. A presentation was given to pupils aged 11–12 years about the study and how they could take part. Recruitment packs were handed out which contained an information sheet about the study, a consent form and a return envelope. Consent forms were signed by both the parent and child and completed forms were returned to school where they were collected by a researcher.

Consenting pupils were assigned a unique ID and given a two-day food diary to complete for the next two consecutive days (i.e. if the diary was handed out on a Monday, they would complete the diary for Tuesday and Wednesday). As with previous study protocol, pupils were asked to write down everything they had to eat and drink from the moment they woke up to the last thing they had before bedtime for both days.

An interview with a researcher was arranged the day after the two recording days. This lasted approximately 40 minutes and was in school time and agreed with the teacher. During the interview, a trained researcher reviewed the food diary with the pupil to clarify information on the foods recorded, such as cooking methods, and to check for commonly forgotten items, such as butter on bread, milk on cereal, and drinks. Pupils were then asked to estimate portion sizes of all food and drink items in the diary using the first method (either 3D food model method (reference method) or Intake24 (test method)). Once the first method was completed, pupils were asked to estimate portion sizes using the second portion estimation method. The order of assessment (i.e. 3D food models first or Intake24 first) was randomised to remove any bias that might arise from the order of administration of the methods.

### Portion estimation using 3D food models (reference method)

**Fig. 1:**
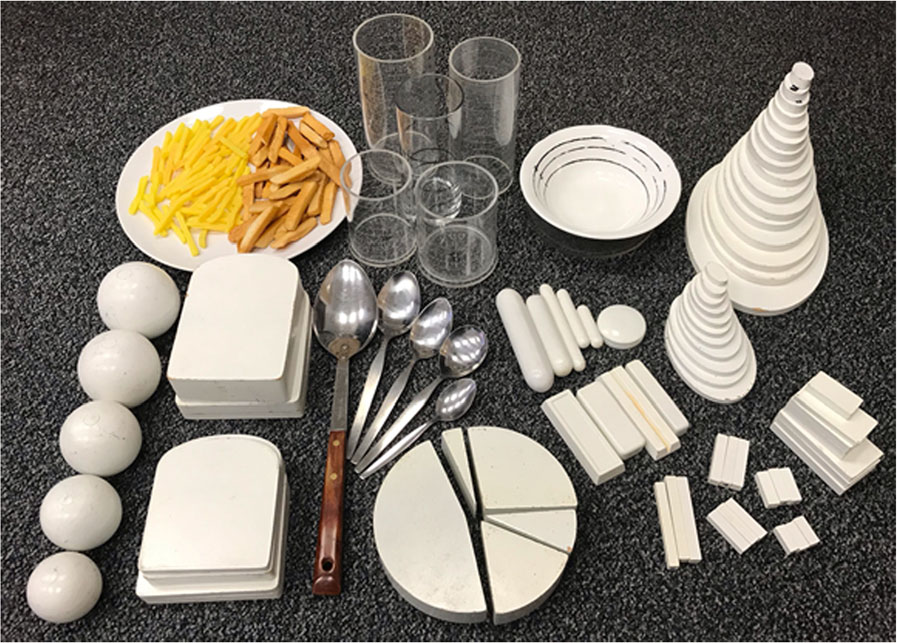
Example of some of the food models used to estimate food and drink portion sizes

Pupils were asked to estimate portion sizes of each food/drink item in the food diary using a range of models on the table in front of them. The models were in the shape of commonly consumed items such as bread, chips, sausages, apples, biscuits (see Table 1 and Fig. 1). They also included spoons, cups, bowls and glasses. A dinner plate was provided so the participant could arrange the models on the plate to help estimate the portion size consumed. The models selected by the pupil were noted in the food diary by the researcher.

**Table 1:** Food models used to estimate food portion sizes in food diary

Model/shape	Range of sizes	Example of types of foods models could be used for
Bread-shaped slices	7 different size slices of differing thicknesses	Bread, lasagne, shepherd’s pie
Sticks	5 different lengths	Carrot sticks, cucumber sticks, green beans, sliced pepper
Chips	Normal cut and French fries	Chips
Spheres	5 sizes	Apples, oranges, potatoes, tomatoes
Pie wedges	12 sizes	Pie, cake, pizza
Sausage-shaped oblongs	5 sizes	Sausages, carrots
Biscuit shapes	10 common sizes/shapes	Biscuits, cake bars
Rectangular oblongs	10 sizes	Cheese slices, cake bars, chocolate
Small oval shapes	11 sizes	Sauces such as ketchup, mayonnaise, mustard, salad cream. Peas, tomato slices, cucumber slices
Large oval shapes	16 sizes	Used to show how much the food covered the plate. Can be piled to show height of food on plate. For foods such as baked beans, pasta sauce, pasta, curry, rice.
Spoons	5 sizes: serving spoon, tablespoon, dessertspoon, soup spoon and teaspoon	Jam, mayonnaise, sauces, vegetables, baked beans, grated cheese
Cups	3 different sizes with graded sides to indicate fill level	Tea, coffee, other drinks
Glasses	5 different sizes with graded sides to indicate fill level	Any drink
Bowls	2 different sizes with graded sides to indicate fill level	Soup, cereals, pasta, rice

All completed food diaries were coded for nutritional composition using the NDNS nutrient databank. This was to allow a direct comparison with Intake24, which uses the same nutrient dataset. Food weights were calculated using conversion factors specific to the food and the food model chosen. For example, the conversion factor for baked beans was 1.15 g/ml. If the volume of the selected model was 34.8 ml, the food weight calculation was 1.15 × 34.8 = 40 g.

Food codes and food weights were entered into a purpose-built Microsoft Access database to allow for data analysis.

### Intake24 (test method)

The process of Intake24 has been reported elsewhere [23]. In brief, the user enters all the foods and drinks consumed the previous day and is then asked to choose the exact food or drink or the closest match from the food list in the system. The user estimates the portion size of the food or drink consumed using portion photographs, before reviewing the final summary of the foods and drinks entered. Intake24 automatically assigns food codes and weights to the foods entered. The data collected in Intake24 is exported as a Microsoft Excel spreadsheet to allow for data analysis.

**Fig. 2:**
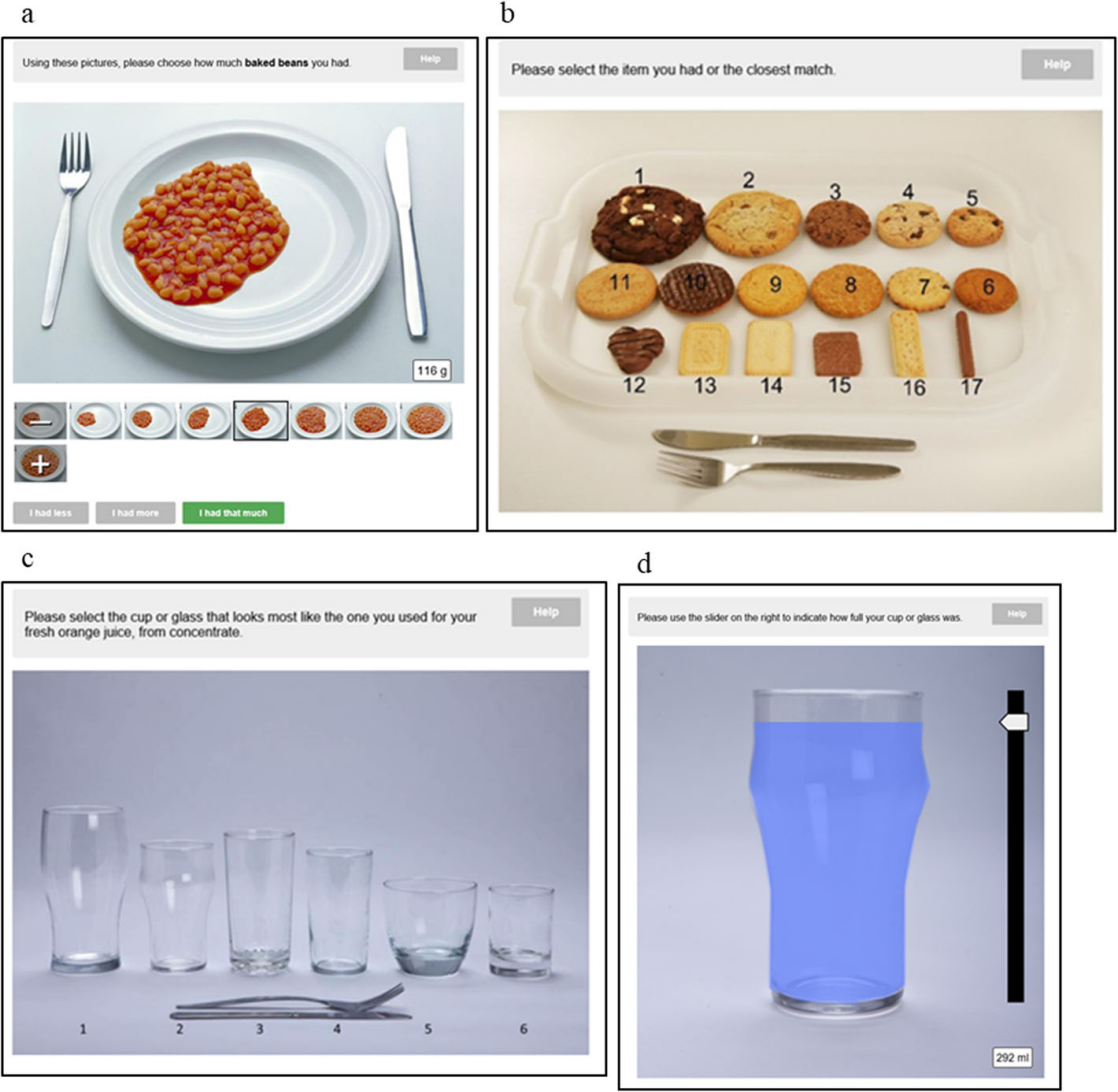
Example of portion estimation photos on Intake24: **a** Increasing serving sizes of baked beans; **b** Guide photo of biscuits; **c** User can select type of glass for drinks; **d** Sliding scale to indicate fill level of glass

In this study, Intake24 was accessed via a study laptop. The researcher entered all the foods and drinks from the food diary into Intake24 and the pupil was asked to estimate the portion sizes for each food and drink by selecting the closest portion photograph on screen. Example portion photos can be seen in Fig. 2. Once the pupil was satisfied that all foods and drinks were entered for both days, the Intake24 entry was submitted.

### Statistical analysis

Mean food (including drinks), energy and nutrient intakes for Intake24 and for the food model method were calculated for all participants completing both methods for the same 2 days. Bland Altman analysis was used to compare the means for each method [24]. The term “food weight” was used to include the weight of foods and drinks.

The Bland Altman method was used to compare the methods and derive limits of agreement [24]. The method calculates that 95% of the differences will lie between the limits of agreement; this is calculated by [23]:

$$ \mathrm{Limits}\ \mathrm{of}\ \mathrm{agreement}=\mathrm{mean}\ \mathrm{difference}\pm 2\mathrm{SD} $$

The smaller the range (between the upper and lower limits), the better the agreement. However, how small this range should be, is dependent on the methods in question and requires meaningful interpretation.

The analyses were performed on logged values, as the data were not normally distributed. The consequence of the log-transform is that the comparison of the reported food weight, energy and nutrient intakes between the methods was assessed by calculating the ratio of an individual’s daily food weight, energy and nutrient intakes estimated using Intake24 to their daily food weight, energy and nutrient intakes estimated using the 3D food models, for each day recorded (2 days in total). A ratio of 1 indicated agreement between the two methods. A ratio of < 1 indicated an underestimation in Intake24 compared to the food model method, and a ratio > 1 indicated an overestimation in Intake24 compared to the food model method. The antilog of the means and ratios were calculated, therefore the results reported uses geometric means and ratios of geometric means [25].

Individual daily average portion sizes for the most commonly consumed food groups were converted into tertiles of intakes (low, medium and high intakes) to account for non-consumers of particular food groups. The numbers of low, medium and high consumers for a particular food group were compared for each method. Statistical analyses were completed in STATA version 15 (StataCorp, College Station, Texas, USA).

## Results

A total of 76 11–12 year olds took part in the study. Six pupils provided incomplete food diaries and/or did not complete Intake24, and were therefore removed from the final dataset. Seventy pupils completed both the food model interview, and Intake24; 64% were female (Table 2). Pupils from all Indices of Multiple Deprivation (IMD) quintiles were represented [26], with more than half (57%) coming from the most deprived quintile.

**Table 2:** Participant demographics for participants completing two-day dietary intake (*n* = 70)

		n (%)
Gender	Male	25 (36)
	Female	45 (64)
IMD quintile^a^	1	15 (21)
	2	2 (3)
	3	8 (11)
	4	5 (7)
	5	40 (57)

^a^1 = least deprived

**Fig. 3:**
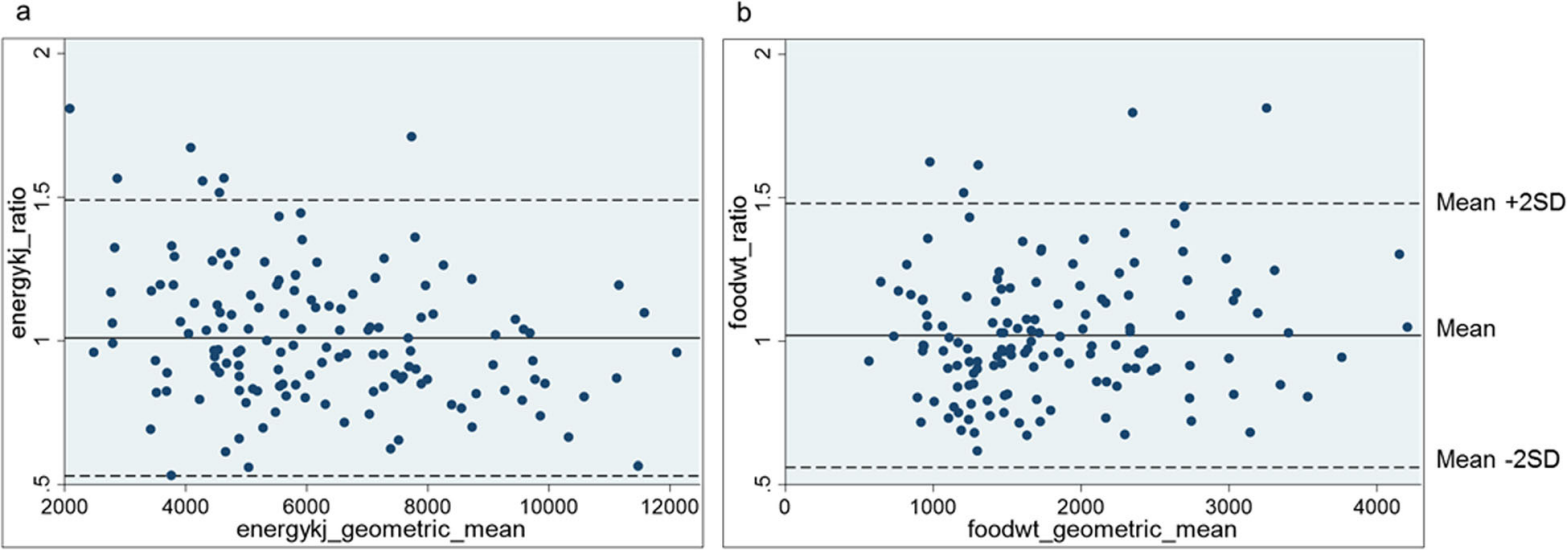
Bland Altman plots of agreement between intakes: **a** agreement in energy intakes (kJ) between Intake24 and 3D food models and (**b**) agreement in food weight (g) between Intake24 and 3D food models

For food weight estimates, the agreement between the two methods is a geometric mean ratio of 1.00, with limits of agreement ranging from minus 35% to plus 53% (Table 3). Intake24 provided estimates of energy intake that were 1% lower on average than estimates of energy intake using the food models, and limits of agreement ranged from minus 38% to plus 57%. Mean intakes of all macro and micronutrients using Intake24 were within 6% of the food model estimates. Limits of agreement were widest for vitamin C (ranging from minus 63% to plus 192%). The Bland Altman plots for energy and food weight show a slight tendency to underestimate energy intakes as the energy (kJ) geometric mean increases (Fig. 3). However, generally there is good agreement in energy and food weight intakes between the methods.

**Table 3:** Agreement of intakes reported using 3D food models and with Intake24, (*n* = 70)

	3D food models		Intake24		Intake24:food models^c^	Limits of agreement	
	Geometric mean	95% CI^b^	Geometric mean	95% CI		lower	upper
Food weight^a^ (g)	1667.7	728.5, 3817.9	1667.1	699.8, 3971.7	1.00	0.65	1.53
Energy (kJ)	5874.1	2635.2, 13,093.8	5800.4	2917.2, 11,533.2	0.99	0.62	1.57
Protein (g)	48.9	18.2, 131.6	47.5	21.6, 104.6	0.97	0.51	1.84
Carbohydrate (g)	190.5	83.8, 433.1	190.8	92.4, 393.9	1.00	0.64	1.57
Total sugars (g)	69.9	22.1, 221.6	71.6	23.3, 219.8	1.02	0.63	1.67
NMES (g)	42.1	8.0, 221.5	43.2	23.3, 219.8	1.06	0.56	2.01
NMES (%)	12.1	2.9, 50.1	12.6	2.6, 62.1	1.08	0.56	2.07
Fat (g)	49.8	17.5, 141.9	49.0	19.0, 126.0	0.98	0.52	1.86
Fat (%)	31.4	18.7, 52.6	31.2	18.6, 52.4	1.00	0.70	1.42
Saturated fat (g)	18.0	5.1, 63.6	17.2	5.3, 56.1	0.96	0.47	1.95
Saturated fat (%)	11.3	5.2, 24.5	11.0	5.0, 24.1	0.97	0.58	1.62
Sodium (mg)	1468.5	476.9, 4522.3	1413.2	508.9, 3924.3	0.96	0.50	1.86
Calcium (mg)	588.3	179.8, 1924.9	558.9	193.7, 1612.7	0.95	0.51	1.77
Iron (mg)	7.7	2.7, 21.6	7.5	2.8, 20.7	0.98	0.56	1.72
Vitamin C (mg)	63.7	8.2, 494.7	66.4	8.9, 496.9	1.04	0.37	2.92

^a^Includes food and drinks; ^b^*CI* Confidence interval; ^c^Ratio of geometric mean

For boys, food weight intakes were 3% lower on average by Intake24 compared to food models, and 2% greater for girls. Similarly, energy intakes were 3% lower on average by Intake24 for boys and there was good agreement for girls (geometric mean ratio 1.00). The greatest difference was seen for vitamin C, with boys’ estimates 7% lower on average in Intake24 compared to food models, and girls’ estimates 11% greater (Table 4).

**Table 4:** Agreement of intakes reported using 3D food models and with Intake24, by gender

	3D Models				Intake24				Intake24:3D food models (limits of agreement)	
	Male (*n* = 25)		Female (*n* = 45)		Male (*n* = 25)		Female (*n* = 45)		Male (*n* = 25)	Female (*n* = 45)
	Geometric mean	95% CI^b^	Geometric mean	95% CI	Geometric mean	95% CI	Geometric mean	95% CI	Ratio of geometric mean (lower; upper)	
Food weight^a^ (g)	1762.8	834.9, 3722.0	1617.1	678.9, 3851.5	1707.1	723.5, 4027.7	1645.3	684.4, 3955.2	0.97 (0.66; 1.41)	1.02 (0.65; 1.59)
Energy (kJ)	6294.1	3184.5, 12,440.2	5653.0	2403.8, 13,294.2	6117.6	3590.8, 10,422.6	5631.4	2644.1, 11,993.6	0.97 (0.58; 1.63)	1.00 (0.65; 1.54)
Protein (g)	52.1	20.1, 134.6	47.3	17.2, 129.7	49.6	23.6, 104.3	46.4	20.6, 104.6	0.95 (0.46; 1.98)	0.98 (0.55; 1.75)
Carbohydrate (g)	207.9	108.2, 399.2	181.5	74.6, 442.0	207.2	118.3, 362.8	182.2	82.7, 401.6	1.00 (0.62; 1.62)	1.00 (0.65; 1.54)
Total sugars (g)	77.6	33.9, 177.7	66.0	18.2, 239.7	78.6	31.5, 196.5	68.0	20.2, 228.7	1.01 (0.58; 1.77)	1.03 (0.66; 1.61)
NMES (g)	46.2	9.7, 219.8	39.9	7.2, 221.8	49.6	9.2, 268.8	39.9	6.1, 259.1	1.07 (0.53; 2.19)	1.05 (0.57; 1.92)
NMES (%)	12.5	2.9, 52.8	11.8	2.9, 49.0	13.8	2.8, 68.0	12.1	2.5, 59.1	1.11 (0.55; 2.23)	1.06 (0.56; 1.99)
Fat (g)	51.7	18.7, 143.3	48.7	16.8, 141.5	49.5	22.0, 111.1	48.7	17.6, 134.7	0.96 (0.47; 1.94)	1.00 (0.55; 1.82)
Fat (%)	30.4	17.3, 53.4	31.9	19.5, 52.0	29.9	17.8, 50.4	32.0	19.2, 53.3	0.98 (0.66; 1.46)	1.00 (0.72; 1.39)
Saturated fat (g)	18.7	5.0, 69.8	17.6	5.1, 60.7	17.5	6.0, 51.3	17.1	4.9, 59.1	0.94 (0.43; 2.03)	0.97 (0.49; 1.91)
Saturated fat (%)	11.0	4.4, 27.4	11.5	5.8, 22.8	10.6	4.9, 22.8	11.2	5.1, 24.9	0.97 (0.57; 1.63)	0.97 (0.58; 1.63)
Sodium (mg)	1685.9	588.2, 4831.9	1360.1	435.2, 4251.2	1539.4	639.8, 3703.7	1347.6	455.0, 3991.8	0.91 (0.43; 1.93)	0.99 (0.54; 1.81)
Calcium (mg)	683.6	263.8, 1771.1	541.2	151.7, 1930.2	630.9	246.5, 1615.0	522.5	173.4, 1574.1	0.92 (0.47; 1.82)	0.97 (0.53; 1.75)
Iron (mg)	8.8	3.9, 19.9	7.1	2.3, 21.6	8.5	3.6, 19.9	7.1	2.4, 20.6	0.96 (0.54; 1.72)	0.99 (0.58; 1.71)
Vitamin C (mg)	71.1	12.7, 397.4	59.9	6.6, 546.9	65.9	8.4, 519.5	66.6	9.1, 490.2	0.93 (0.28; 3.04)	1.11 (0.45; 2.77)

^a^Includes food and drinks; ^b^*CI* Confidence interval

### Food groups

Drinks and fruits and vegetables were the most frequently consumed items. The agreement in average portion sizes of drinks and fruits and vegetables between the two methods can be seen in Tables 5 and 6. In total there were 140 days in which food model estimates and Intake24 data were captured (2 days per pupil). In total, portions of drinks were in the same tertile (low, middle, high) for Intake24 and the food model method on 109 of the 140 days (78%) (39 days for the lowest tertile; 32 days for the middle; and 38 days for the highest tertile) (Table 5). Portions of fruits and vegetables were in the same tertile for Intake24 and the food model method on 111 of the 140 days (79%) (Table 6). The cut-off values for the tertiles were similar for both methods, for drinks and fruit and vegetables. For instance, for drinks, the cut-off value for the lowest tertile for 3D food models was ≤586 g and for Intake24 it was ≤573 g.

**Table 5:** Tertiles of average portion size estimations of drinks for each method, by day

Portion estimations of drinks using 3D food models		Portion estimations of drinks in Intake24			
		Low (≤ 573 g)	Middle (574-1118 g)	High (1119-3755 g)	Total
	Low (≤ 586 g)	39	8	0	47
	Middle (587-1113 g)	7	32	8	47
	High (1114-3525 g)	1	7	38	46
	Total	47	47	46	140

**Table 6:** Tertiles of average portion size estimations of fruit and vegetables for each method, by day

Portion estimations of fruit & vegetables using 3D food models		Portion estimations of fruit & vegetables in Intake24			
		Low (≤ 37 g)	Middle (38-153 g)	High (154-1468 g)	Total
	Low (≤ 30 g)	41	8	0	49
	Middle (31-155 g)	6	32	8	46
	High (156-1625 g)	0	7	38	45
	Total	47	47	46	140

## Discussion

The aim of the study was to compare food weight and nutrient intake estimates for two different methods of portion size estimation; 3D food models (reference method) and digital portion photos on Intake24 (test method). It is important to emphasise that this was a comparison of portion estimation methods specifically, and not of dietary assessment methods. The same two-day food diary was used in both assessment procedures, i.e. the foods and drinks entered in each method were from the same day. The results indicated good agreement in portion size estimations between the two methods, particularly for food weight and carbohydrate. There were underestimations in Intake24 for energy, protein, fat and saturated fat, and overestimations for total sugars and NMES compared with 3D models. However, pupil’s mean intakes using Intake24 were all within 6% of the food model estimates, suggesting overall there was good agreement across all nutrients assessed in this study. Limits of agreement were relatively consistent for all nutrients, with the exception of vitamin C. Substantial differences in the estimated portion sizes of orange juice for Intake24 and the food models for two pupils, potentially explains this finding.

The study compared children’s portion estimates using ‘life-size’ 3D food models with smaller on-screen portion photos in Intake24, and found little difference between the two. Hernandez et al. (2006) reported a similar finding in adults and concluded that factors other than display media, such as the actual serving amount and its closeness in size and shape to the measurement aid, may have more of an effect on the variation in error estimates [27]. There are also additional factors to consider when the focus is on children [28]. The ability to accurately estimate food portion sizes relies upon three main factors; memory, conceptualisation and perception [29] and these are often limited in children. It is largely accepted that from approximately age 10 years, children are able to recall their dietary intake with relative accuracy [30]. However, generally online recall tools are designed to be completed unassisted (without the help of a researcher/nutritionist); this being one of the advantages of using such systems. Intake24 has been tested in users aged 11 years and over [23], however there is evidence that some children under 13 years are unable to accurately complete online dietary recalls unassisted [31, 32]. The ASH11 studies collect dietary data on 11–12 year old pupils, therefore to ensure maximum accuracy in the dietary data collected in the next study, pupils will continue to keep a three-day food diary (consistent with previous study years) and use this as an aid when completing Intake24. The researcher will also be present to assist the pupil in entering the foods and drinks in the diary into Intake24. Pupils will estimate food and drink portion sizes using the on-screen instructions. These approaches will maximise the accuracy of the data collected and enable the benefits of online dietary assessment tools, such as removing the need for manual food coding and increasing the consistency of coding, to be embedded in the dietary assessment protocol for future ASH11 studies.

There are some limitations of the present study. First, the study population is small, and therefore generalisability is limited. Secondly, two-day food diaries were used as the reference method, instead of three-day food diaries, which have been used to collect dietary data in the previous ASH11 studies. Asking pupils to complete a three-day food diary interview followed by three Intake24 recalls would have required a considerable amount of time and motivation for the individual [33]. In view of the age group and the concentration required to complete the tasks, and the time taken out of lessons (approximately 40 minutes), a two-day food diary was considered appropriate for comparisons between the methods.

Intake24 undergoes frequent modifications to ensure the system is fit-for-purpose, for example ensuring food lists, food photos and nutrient information are up to date. To address the key aims of ASH11, study specific questions will be added to Intake24 surveys to allow additional information to be captured, such as, whether a school or home packed lunch was consumed.

## Conclusions

The findings suggest that using the three-day food diary method along with Intake24 for portion size estimation will not impact on the dietary intake data collected, allowing comparisons to be made with data from previous years. The findings show that the bias between the methods is almost always absent, therefore any significant differences in intakes between previous ASH11 surveys and the latest survey, are unlikely to be due to a change in portion size estimation method.

Dietary habits are becoming established in adolescence, and therefore it is crucial that studies, such as ASH11, continue to assess diet but also that methodologies change as technology evolves to ensure that methods are robust but also acceptable to participants. The recruitment of schools to take part in dietary studies is an increasingly difficult task, as they are busy environments with their own time pressures. Methods which can reduce the burden of taking part in such studies and also reduce time needed and financial costs of the research, are of benefit to future dietary assessment with children and in schools.

## Data Availability

The datasets used and/or analysed during the current study are available from the corresponding author on reasonable request.
